# The role of imaging in the management of necrotising enterocolitis: a multispecialist survey and a review of the literature

**DOI:** 10.1007/s00330-018-5362-x

**Published:** 2018-03-26

**Authors:** Margareta Ahle, Hans G. Ringertz, Erika Rubesova

**Affiliations:** 1 Department of Radiology and Department of Medical and Health Sciences, Linköping University, 581 85 Linköping, Sweden; 2 Department of Radiology, Stanford University Medical Center, Stanford, CA 94305 USA; 3 Division of Diagnostic Radiology, Department of Molecular Medicine and Surgery, Karolinska Institutet, Stockholm, Sweden; 4 Department of Radiology, Lucile Packard Children’s Hospital, Stanford University Medical Center, Stanford, CA 94305 USA

**Keywords:** Enterocolitis, necrotising, Abdominal radiography, Ultrasonography, Surveys and questionnaires, Professional practice

## Abstract

**Objectives:**

To investigate current practices and perceptions of imaging in necrotising enterocolitis (NEC) according to involved specialists, put them in the context of current literature, and identify needs for further investigation.

**Methods:**

Two hundred two neonatologists, paediatric surgeons, and radiologists answered a web-based questionnaire about imaging in NEC at their hospitals. The results were descriptively analysed, using proportion estimates with 95% confidence intervals.

**Results:**

There was over 90% agreement on the value of imaging for confirmation of the diagnosis, surveillance, and guidance in decisions on surgery as well as on abdominal radiography as the first-choice modality and the most important radiographic signs. More variation was observed regarding some indications for surgery and the use of some ultrasonographic signs. Fifty-eight per cent stated that ultrasound was used for NEC at their hospital. Examination frequency, often once daily or more but with considerable variations, and projections used in AR were usually decided individually rather than according to fixed schedules. Predicting the need of surgery was regarded more important than formal staging.

**Conclusion:**

Despite great agreement on the purposes of imaging in NEC and the most important radiographic signs of the disease, there was considerable diversity in routines, especially regarding examination frequency and the use of ultrasound. Apart from continuing validation of ultrasound, important objectives for future studies include definition of the supplementary roles of both imaging modalities in relation to other diagnostic parameters and evaluation of various imaging routines in relation to timing of surgery, complications, and mortality rate.

**Key Points:**

• *Imaging is an indispensable tool in the management of necrotising enterocolitis*

• *Predicting the need of surgery is regarded more important than formal staging*

• *There is great consensus on important signs of NEC on abdominal radiography*

• *There is more uncertainty regarding the role of ultrasound*

• *Individualised management is preferred over standardised diagnostic algorithms*

**Electronic supplementary material:**

The online version of this article (10.1007/s00330-018-5362-x) contains supplementary material, which is available to authorized users.

## Introduction

Necrotising enterocolitis (NEC), a potentially devastating intestinal inflammation in neonates, has developed alongside neonatal intensive care [1]. With improving survival of the most premature neonates [2], the epidemiological and pathophysiological landscape keeps shifting [3, 4], and the need to differentiate between NEC of different origins has been pointed out [5–8]. Except for a general agreement that NEC should be differentiated from spontaneous intestinal perforation (SIP) [3, 5, 6, 9, 10], it is not settled whether to aim at a narrower definition of NEC or a sub classification [6–8, 11]. Suggested differential diagnoses include viral enteritis of infancy, feeding intolerance of prematurity, cow milk’s protein allergy, ischaemic bowel disease due to cardiac anomalies, and Hirschsprung’s disease [1, 5, 8]. Factors suggested to aid the differential diagnosis are gestational age (GA), age at onset, feeding volumes, clinical symptoms, stool cultures, blood cultures, and some laboratory tests [1, 5, 12].

Subtle radiographic signs of early NEC may appear before clinical signs and progress ahead of clinical deterioration, but the hallmarks of NEC, pneumatosis intestinalis (PI), and portal venous gas (PVG) are often transient, pneumoperitoneum (PP) frequently missing in spite of intestinal perforation, and the overall sensitivity of abdominal radiography (AR) low, especially in extremely low birth weight infants [4, 9, 13–21]. Based on these insights, together with reports of ultrasound (US) for early detection of PVG [22] and evaluation of mesenteric circulation [23], a standardised algorithm for early diagnosis and evaluation of progress was suggested in 1994. AR in two projections and US was recommended for diagnosis in all cases, followed by repeated examinations at 4-6-h intervals, or at least daily, with supine and left lateral decubitus films each time and repeated ultrasounds every 12 to 24 h [24]. Details of radiographic patterns, analysed already in 1979 to identify early signs of NEC [18], were later systematised in the Duke Abdominal Assessment Scale (DAAS) [25–27].

The staging system, referred to as Bell’s criteria [28, 29], was originally designed as an aid for therapeutic decisions but is widely used to define confirmed NEC as opposed to suspected NEC. Gordon’s suggestions for differentiation between NEC of different origins and other acquired neonatal intestinal diseases (ANIDs) are sometimes referred to as “Gordon’s classification” [5, 20].

Surgical intervention may be considered in deterioration despite medical treatment, indicating intestinal perforation or gangrene [30–33]. Fotter and Sorantin suggested that radiographic and ultrasonographic indications should be “free intraperitoneal gas, free intraperitoneal fluid, and diminished bowel gas with asymmetric loops and persistent dilated loops on at least two follow-up studies” [24]. Of these, only PP on AR is generally accepted [34, 35], but, because of its low sensitivity [9, 13, 36], other signs, such as a persistent/fixed loop are still under discussion [30–32, 37, 38]. PVG on AR has been associated with poor outcome and need for surgery [37–40], mediated by the severity of NEC [41].

In contrast, early reports described PVG on ultrasound as an early sign of NEC [22, 42–44]. Although US was found to be valuable in differentiating NEC and SIP [20], other studies failed to confirm the high sensitivity [45–47] but showed high specificity of most sonographic findings [46, 47]. Doppler studies may show decreased as well as increased mesenteric flow velocities in connection with NEC [48–53].

Complex ascites (focal or echoic fluid) is associated with intestinal gangrene, perforation, the need for surgery, and otherwise poor outcome [16, 19, 37, 54, 55], absent perfusion with bowel necrosis [33, 35], and the finding of a dilated, elongated intestinal loop on AR [56], which is in turn associated with poor outcome [37, 57]. PP detected with ultrasound, as well as various combinations of ultrasonographic signs, may also predict a poor outcome [16].

Despite decades of research, however, the optimal use of imaging in NEC is still unclear. The purpose of this study was to investigate current practices and perceptions regarding imaging in the management of NEC, as described by involved specialists, put them in relation to current literature, and identify issues in need of further discussion.

## Materials and methods

Guided by literature studies, summarised above, interviews with neonatologists and paediatric surgeons, and a pilot survey among paediatric radiologists, a web-based questionnaire on the management of NEC was created in two versions, one for neonatologists and paediatric surgeons and one for radiologists (Appendix 1 and 2). Late complications were not addressed. Links to the questionnaires were distributed by e-mail through European and American specialist organisations for neonatology, paediatric surgery, and paediatric radiology. To increase the number of respondents, passing the links on through personal communication was also accepted.

Two hundred two respondents, 77 neonatologists, 58 paediatric surgeons, and 74 radiologists answered between October 2014 and September 2015. Seven held double specialties in neonatology and paediatric surgery. Nine were general radiologists, of whom five were also specialised in paediatric radiology. To describe areas of agreement and diversity within this multispecialist group as a whole, the results were analysed with proportion estimation with 95% confidence intervals (CI). Differences between specialities were also noted and regarded as significant if the CIs did not overlap. In these cases, *p* values were obtained by chi^2^ tests, and analyses were repeated without the dominant groups of radiologists from the Americas (*n* = 48) and neonatologists from the British islands (*n* = 30), attempting to discern whether the differences were more likely due to geographical variations in clinical traditions or diverging perceptions between specialties. The results of such supplementary analyses are provided in the text. Details of distribution between countries and specialties are given in supplementary table S1.

## Results

### Differential diagnosis

Paralytic ileus in sepsis was regarded the most important differential diagnosis, closely followed by SIP. There were some significant differences between specialties in the invariable consideration of certain diagnoses. See Table 1.

**Table 1.** Differential diagnosis

Differential diagnosis								
Considered								
			Sometimes			Always		
			*n* [%]	(95% CI)	*p*	*n* [%]	(95% CI)	*p*
Paralytic ilieus in sepsis, *n* = 202			65 [32%]			127 [63%]		
	Neonatologists, *n* = 77		15 [19%]	(12–30%)		59 [77%]	(66–85%)	
	Paediatric surgeons, n=58		18 [31%]			37 [64%]		
	Radiologists, *n* = 74		33 [45%]	(33–56%)	0.001	37 [50%]	(38–62%)	0.001
Spontaneous intestinal perforation, *n* = 202			83 [41%]			105 [52%]		
	Neonatologists, *n* = 77		28 [36%]			47 [61%]	(50–73%)	
	Paediatric surgeons, *n* = 58		21 [36%]			34 [59%]		
	Radiologists, *n* = 74		36 [49%]			28 [38%]	(27–49%)	0.011
Feeding intolerance of the premature, *n* = 128^a^			47 [37%]			63 [49%]		
	Neonatologists, *n* = 77		24 [31%]			46 [60%]	(48–70%)	
	Paediatric surgeons, *n* = 58		25 [43%]			18 [31%]	(20–44%)	< 0.001
Gastrointestinal malformation, *n* = 202			125 [62%]			46 [23%]		
Ileus from meconium or other obstruction, *n* = 202			111 [55%]			59 [29%]		
Hirschsprung's disease, *n* = 202			123 [61%]			32 [16%]		
Viral enteritis/gastroenteritis, *n* = 202			95 [47%]			20 [10%]		
	Neonatologists, *n* = 77		49 [64%]	(53–76%)		1 [1%]	(-1–4%)	
	Paediatric surgeons, *n* = 58		25 [43%]			3 [5%]	(-1–11%)	
	Radiologists, *n* = 74		25 [34%]	(24–46%)	< 0.001	18 [24%]	(15–35%)	< 0.001
Cow milk protein allergy, *n* = 128^a^			63 [49%]			4 [3%]		

^a)^In clinicians’ questionnaire only

Percentages refer to the proportions of respondents. Where significant differences between specialties were detected, 95% confidence intervals and *p* values are given. Supplementary analyses showed no substantial influence of geographical variations

Other suggested differential diagnoses: tympanism due to CPAP (3); hypoperfusion/circulatory insufficiency, e.g. due to congenital heart disease (2); immature gastrointestinal motility (2); paralytic ileus due to other causes than sepsis (metabolic, hypokalaemia, hypothyroidism, narcotics ) (3); incarcerated hernia (1); obstipation (1); eosinophilic proctocolitis (1). Cow milk protein allergy was stated in free text by one radiologist

The most important aspects, influencing the differential diagnosis, were the clinical picture, radiographic findings (clinicians only), degree of prematurity, and age at onset; see supplementary table S2.

### Classification

A total of 75%, 51% (40-63%) of radiologists and 88% (81-93%) of clinicians (CI within brackets), *p* < 0.001, reported that they always used some classification of NEC and 15% (8-25%) of radiologists and 1.6% (0.3-6%) of clinicians that they never do, *p* < 0.001. An assessment of “suspected”, “definite medical”, or “surgical” NEC was used, at least sometimes, by 88 %, variants of Bell’s criteria by 59 %, and Gordon’s classification by 8 %. The differences were independent of the geographical locations of the hospitals.

The DAAS, mentioned in three free comments, was not included among the response alternatives since it is a system for evaluation of AR rather than a NEC classification.

### Use of imaging

**Fig. 1.**
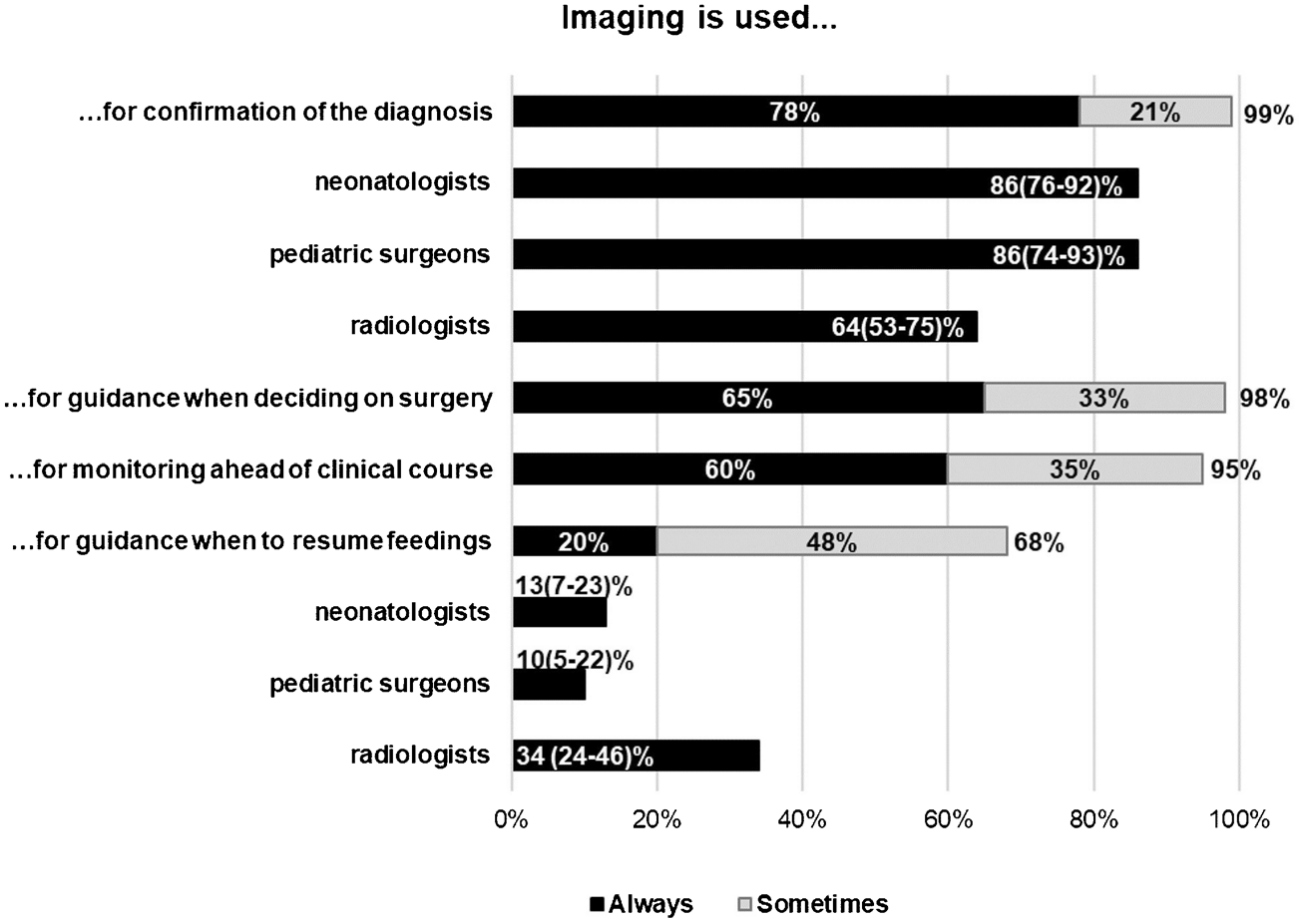
Use of imaging. Percentages refer to proportions of the total number of 202 respondents. Where significant differences were detected, percentages for the subgroups of 77 neonatologists, 58 paediatric surgeons, and 74 radiologists are presented with 95% confidence intervals within brackets. For the invariable use of imaging for confirmation of the diagnosis, *p* = 0.002, and before resuming feedings, *p* = 0.001. Supplementary analyses showed no influence of geographical differences on the use of imaging for confirmation of the diagnosis but on the use of imaging before resuming feeding

The most common use of imaging was for confirmation of the diagnosis. According to significantly more neonatologists than radiologists, imaging was always used for this purpose (Fig. 1). The difference was not explained by geographical differences, which, in contrast, seemed to influence the use of imaging before resuming feeding.

The choice of modality was most often made by clinicians, but radiologists frequently reported that they were involved in the decision, and, according to 65 % of all of them, radiologists and clinicians decided by consent at least sometimes. There was geographical variation in this respect, most likely reflecting differences in clinical tradition.

### Use of abdominal radiography

**Fig. 2.**
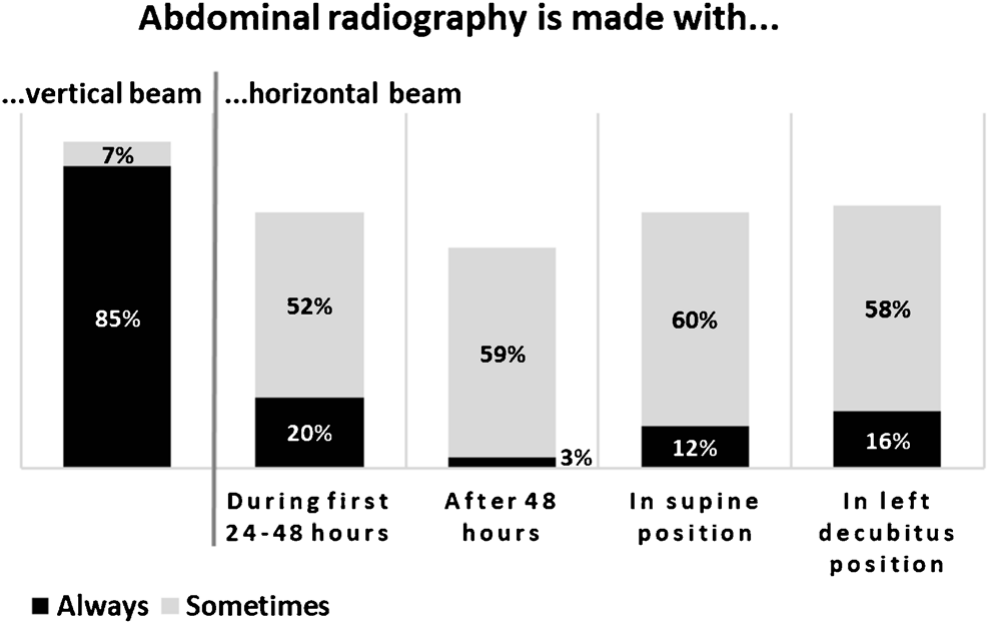
Projections in AR. Reported use of AR in suspected or known NEC. The left column represents the use of radiography with a vertical beam. The four columns to the right represent the use of a horizontal beam regarding time course and preferred patient positions. Percentages refer to proportions of the total number of 202 respondents

AR was the modality most widely used as first choice, “always” stated by 92 % and “sometimes” by another 7 %; see supplementary fig. S1. The use of projections is summarised in Fig. 2. AR with a vertical beam was commonly used in all patients with suspected NEC, but for the horizontal beam, there was no uniform routine and no clear-cut preference for the supine or left decubitus position.

Ninety-one per cent (82-96%) of neonatologists and 83% (70-91%) of paediatric surgeons stated that they always read AR by themselves, whereas only 18% (10-28%) of radiologists did so, *p* < 0.001.

The reported importance of the findings on AR are summarised in Table 2. The last column contains the corresponding DAAS score. The findings considered most important were PP and PI, followed by PVG and “fixed loop” on sequential radiographs.

**Table 2.** Importance of findings on AR

Importance of findings on AR, *n* = 202			
	Importance *n* [%]		
	Some	Great	DAAS^a^
Pneumoperitoneum/free gas	4 [2%]	196 [97%]	10
Pneumatosis intestinalis/intramural gas	6 [3%]	194 [96%]	6; 8
Portal venous gas	22 [11%]	180 [89%]	9
Persistent loop on sequential radiographs	59 [29%]	139 [69%]	7
Pattern of gas distribution	99 [49%]	99 [49%]	0–3
Intestinal dilatation	97 [48%]	95 [47%]	1–3
Separation of intestinal loops	113 [56%]	71 [35%]	4–5

^a)^Duke Abdominal Assessment Scale [25]

Percentages refer to the proportions of all 202 respondents. No significant differences between subgroups were detected

Findings suggested in free text: “grey abdomen” (1), ascites (2), and thickened bowel wall (1). The first may correspond to featureless or multiple separated bowel loops, i.e. 5p on the DAAS, the latter two to separation of intestinal loops

### Use of ultrasound

**Fig. 3.**
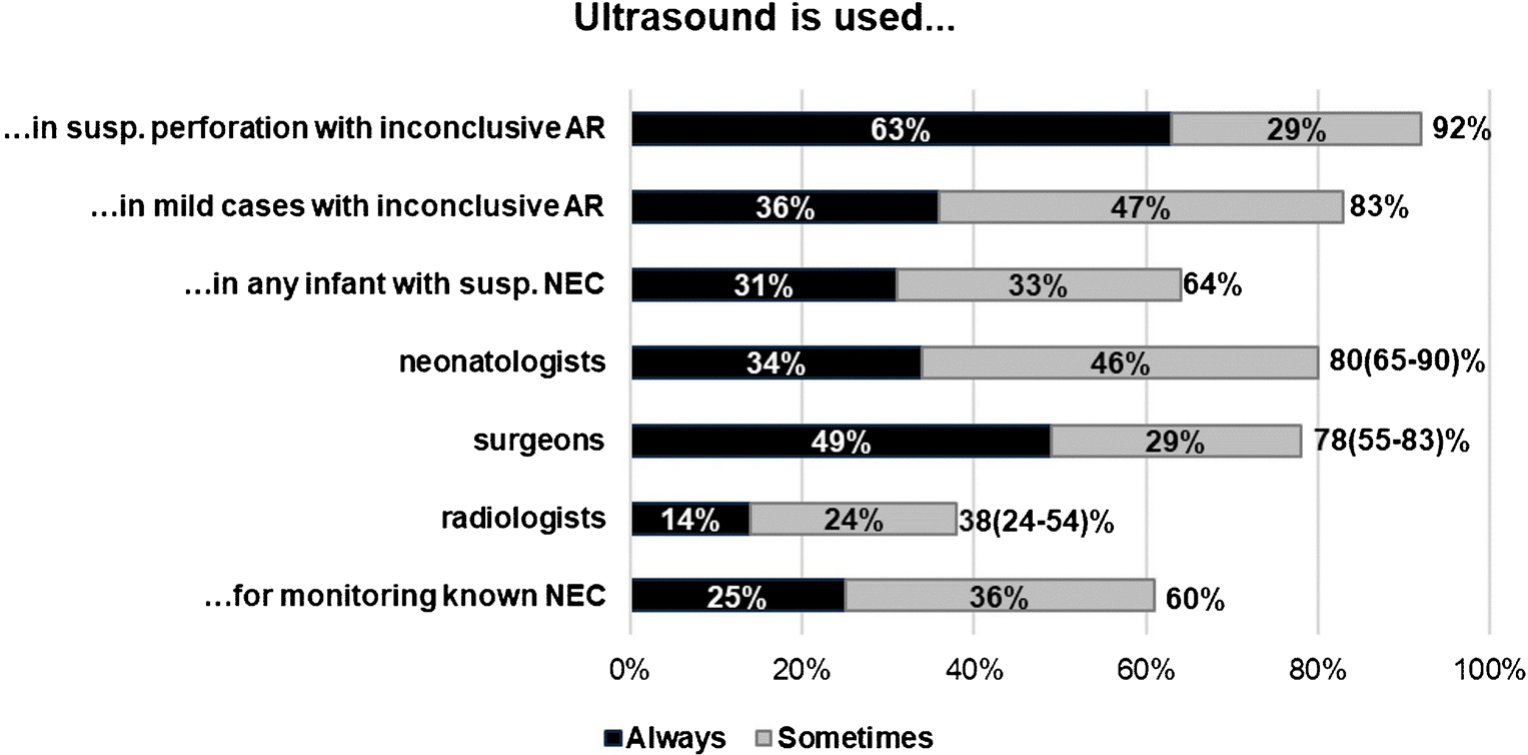
Use of ultrasound. Use of ultrasound according to the 118 respondents from hospitals where ultrasound was done for NEC, 41 neonatologists, 41 paediatric surgeons, and 42 radiologists. Six held double specialties in neonatology and paediatric surgery. Percentages refer to the proportion of respondents. The totals of positive responses, sometimes and always, are given at the right side of the bars. Where significant differences between specialties were found, 95% confidence intervals are given within brackets, *p* < 0.001. The difference was levelled out when American radiologists were excluded, indicating that it was conditioned by geographical variations in clinical traditions rather than diverging perceptions between specialties

Fifty-eight per cent without significant differences between specialties or regions stated that ultrasound was used for NEC at their hospital. Ninety-three per cent of these would, at least sometimes, combine AR and US, and 52% might sometimes use US as first choice. The most common use of US was in patients with inconclusive AR, especially in severe cases with suspected but not verified perforation (Fig. 3).

Opinions about US regarding usefulness, availability, time consumption, and inconvenience for the patient were more diverse than the understanding of findings in AR, the general perception being more positive among respondents from hospitals where ultrasound is used for NEC, with differences between specialties concerning time consumption. Details are given in Table 3.

**Table 3.** Opinions about ultrasound in NEC

Opinions about ultrasound in NEC, *n* = 202				
Ultrasound in NEC…	Sometimes n [%]	Yes n [%] (95% CI)		*p*
…is useful	75 [37%]	99 [49%]		
…is readily available	59 [29%]	85 [42%]		
…is time-consuming	59 [29%]	42 [21%]		
Neonatologists, *n* = 77		5 [6%]	(3 – 15%)	
Surgeons, *n* = 58		8 [14%]	(7 – 26%)	
Radiologists, *n* = 74		31 [42%]	(31 – 54%)	< 0.001
…disturbs the infant	81 [40%]	8 [4%]		
…should be used more	46 [23%]	97 [48%]		

Response alternatives for each statement were "yes", "sometimes", "no" and "no opinion"

Percentages refer to proportions of respondents. Where significant differences between subgroups were detected, 95% confidence intervals and a *p* value are given. Supplementary analyses showed no substantial influence of geographical variations

Comments about ultrasound were: “painful” (1); “has not been done at my institution/no experience/US is not used as a routine imaging modality in our hospital/department” (3); “it is not always possible at 24 hours” (1); “depends strongly on experience of the operator” (1); “need more training for sonographers and radiologists in US for NEC” (1); “not really available in our unit—would like it to be” (1); “not yet used as experience in detecting pneumatosis, etc., is lacking, except for general assessment” (1); “doing US in NEC for 15 years” (1); “we use it very frequently already—as often as needed” (1); “very useful and valid in experienced hands” (1)

The most frequently evaluated signs on ultrasound were focal fluid collections, PVG, echoic fluid, and thickening of the intestinal wall. Details are given in Table 4.

**Table 4.** Signs looked for on US

Signs looked for with ultrasound, *n* = 118	Evaluated by ^a^ *n* [%]	Reported association with adverse outcome ^b^
Focal fluid collections	111 [94%]	A[16, 37, 57]
Turbid/echoic fluid	109 [92%]	A[19, 37, 54, 57], B[16]
Portal venous gas	109 [92%]	B[16]
Bowel wall thickening	107 [91%]	A[37, 57], B[16]
Pneumatosis intestinalis/intramural gas	101 [86%]	B[16]
Clear/anechoic fluid	101 [86%]	
Pneumoperitoneum/free gas	93 [79%]	A[16]
Intestinal motility	92 [78%]	A[37], B[33]
Bowel wall perfusion (with Doppler)	76 [64%]	B[16, 33, 35]
Mesenteric circulation (with Doppler)	68 [58%]	
Bowel wall thinning	67 [57%]	B[35]

^a)^Percentages refer to respondents from hospitals where ultrasound is done for NEC, *n* = 118 respondents: 41 neonatologists, 41 paediatric surgeons, and 42 radiologists. Six held double specialties in neonatology and paediatric surgery. No significant differences between specialties were observed. ^b)^ Adverse outcome such as need for surgical intervention or death, associations as reported in the literature. References within square brackets

A. Independently associated with adverse outcome

B. Associated with adverse outcome if present together with other signs

As for intestinal motility and bowel wall perfusion, reported associations refer to *reduced* motility and *absent* perfusion

Signs stated in free text were echogenicity of the intestinal wall, bowel motility, amount and location of fluid collections, and “zebra sign”, all of which are thought to be covered by the response alternatives above

Fourteen per cent of neonatologists stated that all clinicians at their department do abdominal US and 32% that some do. Where at least some clinicians performed US, 47% had support from the radiology department in the evaluation of the results—pictures (18%), cine loops (12%), or both (18%).

Fifty-one per cent (40-63%) of radiologists, compared to 20% (14-28%) of clinicians, rejected the idea that clinicians who do not do ultrasound should learn it, *p* < 0.001.

### Repeated imaging

**Fig. 4.**
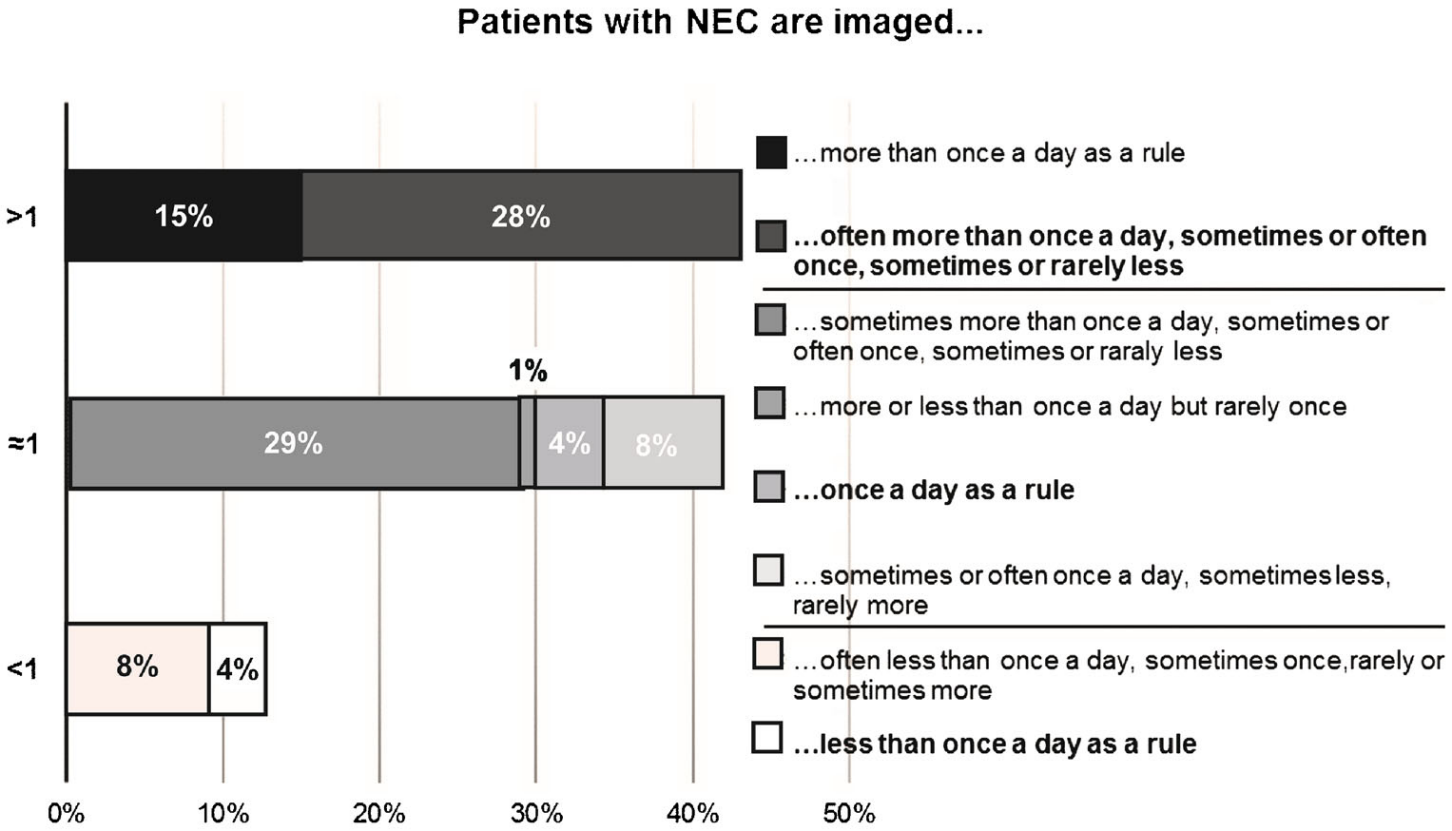
Examination frequency. For each of three suggested frequencies, “more than once every 24 h”, “about every 24 h”, and “less than every 24 h”, respondents could choose response alternatives “often”, “sometimes”, or “rarely”. The diagram summarises the distribution of response combinations among all 202 respondents

There was considerable variation in examination frequency; see Fig. 4.

The frequency was usually decided individually, but 61% sometimes used a fixed schedule, and 10% always did. Sixty-three per cent stated that radiation might be a concern.

Whether NEC was confirmed or not, around 90 % would at least sometimes use the same modality for repeated imaging. See supplementary Table S3.

### Indications for surgery

PP on AR was regarded an indication for surgery, at least sometimes, by 99% and clinical deterioration despite medical treatment by 96% of clinicians; see Table 5.

**Table 5.** Indications for surgery

	Indications for surgery					
	Sometimes			Always		
	n [%]	(95% CI)	*p*	n [%]	(95% CI)	*p*
Pneumoperitoneum on AR, *n* = 202	22 [11%]	(7–16%)		178 [88%]	(82–92%)	
Pneumoperitoneum on US … all, *n* = 202	51 [25%]	(20–32%)	< 0.001^e^	101 [50%]	(43–57%)	< 0.001^e^
… where US was used for NEC, *n* = 118	37 [31%]	(23–40%)		72 [61%]	(52–69%)	
… where US was not known to be used for NEC, *n* = 84	15 [18%]	(11–28%)		29 [35%]	(25–45%)	0.037^f^
Portal venous gas on AR, *n* = 202	93 [46%]			38 [19%]		
Portal venous gas on US^a^ … all, *n* = 202	79 [39%]			22 [11%]		
… where US was used for NEC, *n* = 118	54 [46%]			15 [13%]		
… where US was not known to be used for NEC, *n* = 84	25 [30%]			7 [8%]		
Fixed/persistent loop on sequential AR, *n* = 202^b^	121 [60%]			30 [15%]		
Turbid or localized fluid on US^c^ … all, *n* = 202	114 [56%]			24 [12%]		
… where US was used for NEC, *n* = 118	80 [68%]	(59–76%)		18 [15%]	(10–23%)	
… where US was not known to be used for NEC, *n* = 84	34 [40%]	(30–51%)	< 0.001^f^	6 [7%]	(3–15%)	
Clinical deterioration despite medical treatment, *n* = 128^d^	52 [41%]			72 [56%]		

Ninety-five per cent confidence intervals and *p* values are specified where there were significant differences between modalities or respondents with and without experience of US in NEC

^a)^A significantly greater proportion of surgeons than radiologists regarded portal venous gas on ultrasound as “sometimes” an indication for surgery: 52% (39-65%) vs. 26% (17-37%), *p* = 0.002. Summarising all positive responses (always and sometimes), the difference was significant between PVG on AR, 65% (58-72%), and on US 50% (43-57%), *p* = 0.003

^b)^A significantly greater proportion of radiologists than surgeons rejected persistent loop as an indication for surgery: 31% (21-43%) vs. 7% (3-17%), *p* = 0.001. Supplementary analyses showed no substantial influence of geographical variations

^c)^A significantly greater proportion of neonatologists than radiologists rejected turbid or localised fluid as an indication for surgery: 36% (26-48%) vs. 9% (4-19%), *p* < 0.001. There was no substantial influence of the geographical locations of the hospitals of the respondents

^d)^Included in the clinicians' questionnaire only

^e)^PP on AR compared to PP on US

^f)^Where ultrasound was known to be used in NEC compared to where it was not

### What is the role of imaging in NEC management and what should it be?

This was an open question to obtain a perception of our respondents’ expectations from imaging in the current management of NEC and a desirable development. One vision expressed was that imaging should be non-invasive, easy to perform, and easily repeatable, another that it should be bedside. Radiologists as well as clinicians repeated the importance of imaging in the management of NEC, some stressing its importance in surveillance over diagnosis, a few its role in the differential diagnosis. Identification of late complications was mentioned. Especially clinicians, but also a few radiologists, pointed out that imaging should always be used, interpreted, and planned in the context of clinical evaluation, and some highlighted the importance of close interaction among radiologists, neonatologists, and paediatric surgeons in this regard. There were also critical remarks about excessive use of X-rays. Around 30 per cent of comments, from radiologists and clinicians alike, referred to the use of US. Remarks about an observed or desired increase in its use for NEC were more common than statements that it would not add much to AR. Another frequent comment was that ultrasound should be used for problem solving rather than routinely, but the use of ultrasound for monitoring was also suggested. Some radiologists pointed out the lack of resources as an obstacle to using US, and some clinicians noted the need for more scientific validation of the method. Different traditions and opinions regarding ultrasound in the hands of clinicians shine through in occasional answers.

## Discussion

Thanks to easy distribution of web-based questionnaires, this survey collected many respondents , evenly distributed between specialties but, unfortunately, with uneven geographical distribution within specialties, partly impairing a reliable distinction between influences of specialty and regional variation on the responses. With the open invitation, the real reach of the survey could not be estimated and no response rate could be calculated. The respondents thus represent primarily themselves: 202 professionals, enough involved in the management of NEC to be reached by the questionnaire and respond to it. Consensus can be expected to be greater in this group than among unselected neonatologists, paediatric surgeons, and radiologists, which is possibly further enhanced by accepting a minor contribution of questionnaires distributed by personal communication. The special interest may explain the almost 60% reported use of US in NEC. Keeping the selection of respondents in mind, however, the responses should be useful as a base for discussion about desirable studies and future guidelines.

Despite decades of efforts to improve diagnostic accuracy in NEC, there is no generally accepted routine, and the role of imaging in relation to new methods of surveillance has not been defined.

The usefulness of imaging for confirmation of the diagnosis, decisions on surgery, and surveillance is uncontroversial among the respondents to this survey. Imaging before resuming feeding seems to be an established practice at some centres.

The findings almost unanimously perceived as most important on AR correspond well to the upper scores of the DAAS (Table 2).

The lack of a standardised diagnostic algorithm may correspond to the broad spectrum of different presentations and courses of NEC. The suggested frequency of AR every 6th hour to avoid delay of adequate treatment was motivated by the transient nature of radiographic signs, sometimes preceding clinical deterioration. Although proposed in 1994 [24], US is rarely used as a part of the routine work-up but rather for gathering more information when AR is inconclusive. Suggested applications are early detection of PVG, evaluation of fluid in the abdominal cavity, and assessment of bowel wall perfusion [58], of which the former two are frequently used according to our respondents. US for NEC surveillance, as suggested by some respondents, was less common, despite the absence of radiation and possible high sensitivity of some ultrasonographic signs for the need of surgery [16, 19, 33, 35, 54, 57].

Concern about radiation was an issue of low agreement among our respondents. Scott et al. found a risk of total radiation exposure exceeding a preferred limit of 1 mSv in infants in neonatal intensive care [59]. Whether the use of US lowers the frequency of AR cannot be discerned from our results. Studies investigating the impact of imaging frequency and choice of modality for surveillance on parameters such as timing of surgical intervention and complication rates would be helpful to minimise radiation while maintaining the best possible results.

For the differential diagnosis, 75% of responding clinicians would attribute at least some importance to US—a greater proportion than have access to it. Nevertheless, 22% stated that it would be of no importance at all. The value and reliability of ultrasound in NEC remains controversial, but among respondents with experience of US in NEC attitudes seem more positive.

Although most survey respondents thought that US would, at least sometimes, be useful, the often repeated objections of availability and operator dependency [60] were expressed, as were concerns about the validity of ultrasonographic findings. As for operator dependency, the importance of good technique obtaining the pictures and the interobserver variability at interpretation of AR should not be ignored [21, 61, 62]. Saving pictures, especially cine loops, together with a systematic approach enables re-evaluation of ultrasonographic examinations and may reduce operator dependency.

Validation studies are difficult to design because of the “lack of a robust gold standard” [46] apart from findings at surgery or autopsy. This problem applies also to AR, which, however, is more established in the clinical tradition. Many recent studies use outcome as reference standard, concentrating on the role of imaging for monitoring and decision on surgery. This role, which goes well with the priority of our respondents to distinguish NEC that needs surgery from less serious NEC and NEC-like conditions, may increase if infants at high risk of developing surgical NEC are identified to a greater extent by biomarkers or near-infrared spectroscopy (NIRS) [34, 63].

In the differential diagnosis, the clinical picture was almost unanimously regarded as the most important aspect, and it is well known that suspected NEC patients are usually treated for NEC, regardless of any formal criteria for diagnosis and staging, and may even be operated on if their clinical state is severe enough [9, 24, 41, 64, 65].

The decision on surgery is a balancing act between early intervention to prevent progression of disease and avoidance of unnecessary surgery, with considerable variation in the readiness to intervene. One respondent commented that many babies tolerate a pneumoperitoneum very well, but the opinion that surgical management becomes necessary in persistent PI at 48 h was also expressed. Even if imaging is perceived to be important for confirming the diagnosis, its most important role may be for the timing of surgery, and reported results on the potential of US to bridge the lack of sensitivity of AR for intestinal perforation and bowel necrosis are promising [33, 54, 56, 57].

The ability of US to detect smaller amounts of intraperitoneal gas, presumably from contained or self-limiting perforations, may partly explain that respondents perceived PP differently when detected with US than on AR. In contrast, complex ascites is also indicative of perforation and could be expected to prompt surgery more often than reported. Experience of ultrasound in NEC seems to influence the evaluation of US signs.

The distribution of opinions on persistent loop on sequential radiographs and portal venous gas on AR as indications for surgery is similar to the equivocal results in the literature [30–32, 37–41].

Regarding surgery, PVG was evaluated similarly on AR and US, in contrast to the concept of PVG on US as an early sign of NEC. The latter may rely on an over-interpretation of the first reports [22, 42–44] or be influenced by altered compositions of the patient population, extremely premature infants being less likely to present with PI and PVG [41, 46, 66]. In 15 infants reported in 1986, mean GA was 33 weeks [44] compared to 26 weeks median GA for 25 patients reported 30 years later [54].

Differences between specialties must be interpreted with care, since, even where supplementary analyses did not reveal any substantial regional influence, practices may vary between hospitals within the same region, and most respondents did not work at the same hospital. Perspectives on the differential diagnosis varying according to field of expertise could be expected. How time consumption of ultrasound is perceived may depend on whether it is compared to other clinical bedside examinations or to obtaining an AR.

Clinicians evaluating radiographic images, or doing ultrasound, do not eliminate the need for radiological expertise but might contribute to a deeper understanding of the results and facilitate the close interaction between involved specialists, called for by some respondents. Radiologists need to get clinically involved, be aware of the consequences of reported signs, and discuss their implications with clinicians. Understanding the influence of the pathophysiological dynamic of NEC and its variation with gestational age on radiographic and ultrasonographic findings is important to tailor imaging according to the needs of the individual patient. The choice of modality as well as the examination schedule should ideally be decided by consent. US and Doppler may, however, confidently be recommended, at least for evaluation of the need for surgery.

Together with the clinical picture, imaging is an indispensable tool in the management of NEC: for detecting complications where the diagnosis is already established and solving the diagnostic puzzle where it is not. This survey shows great agreement on the most important signs of NEC on AR and their significance, with more than 90% agreement independent of specialty, but considerable diversity in imaging routines. Individualised management is preferred over standardised algorithms. For US, there is more variability in perceptions, partly depending on the respondents’ experience of US in NEC. Apart from further validation of ultrasound in the various stages of NEC, future studies should seek to define the supplementary roles of both imaging modalities in relation to other diagnostic parameters such as biomarkers and NIRS and to evaluate imaging routines in relation to timing of necessary interventions, occurrence of complications, and mortality rate. Future guidelines should probably focus on evidence-based support for individualised decisions rather than uniform protocols to fit all.

## Electronic supplementary material

ESM 1 (DOCX 197 kb)

ESM 2 (PDF 137 kb)

ESM 3 (PDF 166 kb)

## Abbreviations

ANID: Acquired neonatal intestinal disease
AR: Abdominal radiography
CI: Confidence interval
DAAS: Duke abdominal assessment scale
GA: Gestational age
NEC: Necrotising enterocolitis
NIRS: Near infra-red spectroscopy
PI: Pneumatosis intestinalis
PP: Pneumoperitoneum
PVG: Portal venous gas
SIP: Spontaneous intestinal perforation
US: Ultrasound
