# Supplementary material for: The role of imaging in the management of necrotising enterocolitis: a multispecialist survey and a review of the literature
Source: Eur Radiol. 2018 Mar 26;28(9):3621–31. doi: 10.1007/s00330-018-5362-x (PMC6096607; doi:10.1007/s00330-018-5362-x)
Supplement: Supplementary file 1 — (DOCX 197 kb) [file 330_2018_5362_MOESM1_ESM.docx]

**
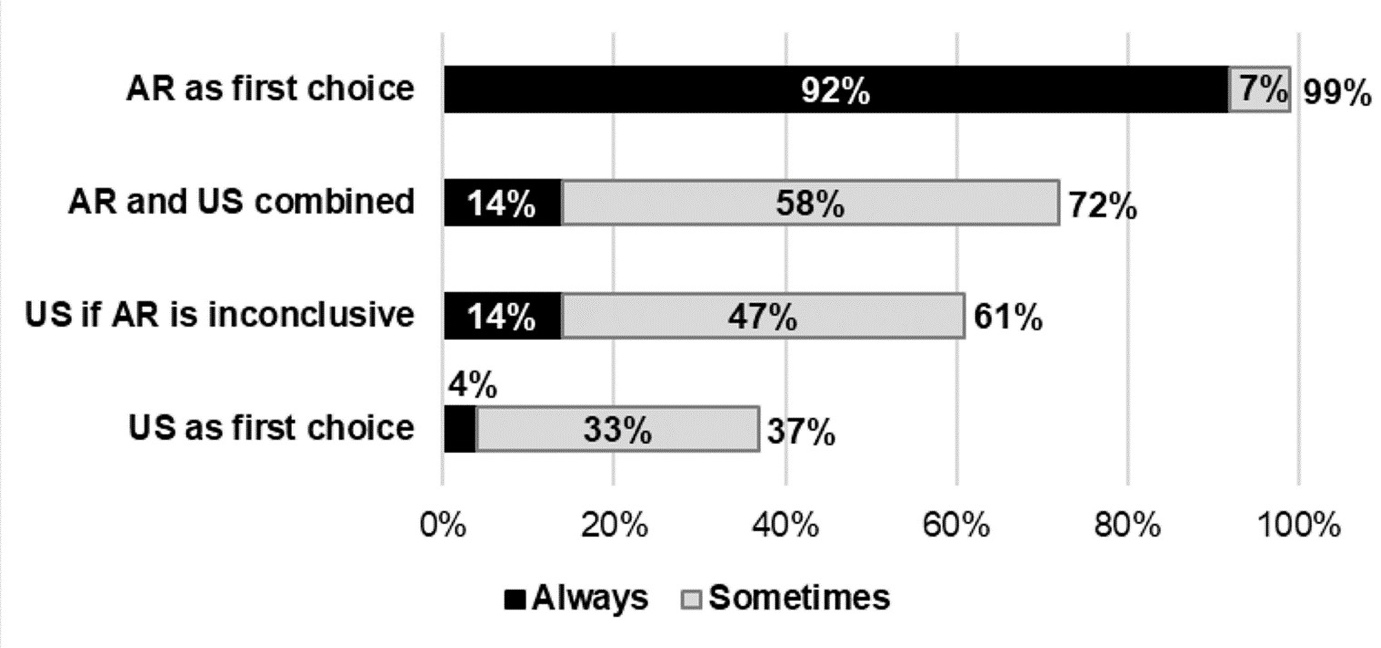
Supplementary fig S1. Choice of modality**

Percentages refer to the proportion of all 202 respondents. The totals of positive responses, sometimes and always, are given at the right side of the bars. Radiologists were also asked if they used an individualised approach, to which 65% answered that they did: 45% sometimes, 20% always.

**Table S1:** **Distribution of respondents between countries and specialties**
^a^Double refers to double specialties in neonatology and paediatric surgery.
^b^9 radiologists were general radiologists, of whom 5 (1 from Oman, 3 from Sweden, and 1 from the USA) were also specialised in paediatric radiology.

| Distribution of respondents between countries and specialties | | | | | | |
| --- | --- | --- | --- | --- | --- | --- |
| Country | Radiologists | Neonatologists | Surgeons | | Double^a^ | Total |
| Australia | 1 | 3 | | 0 | 0 | 4 |
| Austria | 1 | 1 | | 1 | 0 | 3 |
| Belgium | 0 | 0 | | 1 | 0 | 1 |
| Bulgaria | 0 | 1 | | 1 | 0 | 2 |
| Canada | 8 | 1 | | 0 | 0 | 9 |
| Chile | 1 | 0 | | 0 | 0 | 1 |
| Czech Republic | 1 | 0 | | 0 | 1 | 2 |
| Denmark | 0 | 4 | | 0 | 0 | 4 |
| Egypt | 0 | 0 | | 1 | 0 | 1 |
| Finland | 1 | 0 | | 1 | 0 | 2 |
| France | 0 | 1 | | 4 | 2 | 7 |
| Germany | 0 | 4 | | 1 | 1 | 6 |
| Greece | 0 | 0 | | 1 | 0 | 1 |
| Guernsey | 0 | 1 | | 0 | 0 | 1 |
| Hungary | 0 | 0 | | 3 | 0 | 3 |
| Ireland | 0 | 4 | | 1 | 0 | 5 |
| Israel | 0 | 3 | | 2 | 0 | 5 |
| Italy | 0 | 1 | | 5 | 0 | 6 |
| Japan | 0 | 1 | | 0 | 0 | 1 |
| Kenya | 1 | 0 | | 0 | 0 | 1 |
| Latvia | 0 | 0 | | 2 | 0 | 2 |
| Mexico | 0 | 0 | | 1 | 0 | 1 |
| Netherlands | 2 | 5 | | 2 | 0 | 9 |
| New Zealand | 1 | 1 | | 0 | 0 | 2 |
| Norway | 0 | 0 | | 1 | 0 | 1 |
| Oman | 1^b^ | 0 | | 0 | 0 | 1 |
| Philippines | 1 | 0 | | 0 | 0 | 1 |
| Poland | 0 | 0 | | 1 | 0 | 1 |
| Portugal | 0 | 0 | | 2 | 1 | 3 |
| Romania | 0 | 0 | | 3 | 0 | 3 |
| Saudi Arabia | 0 | 0 | | 2 | 1 | 3 |
| Serbia | 0 | 0 | | 0 | 1 | 1 |
| Slovenia | 1^b^ | 0 | | 1 | 0 | 2 |
| South Korea | 1 | 0 | | 1 | 0 | 2 |
| Spain | 1 | 3 | | 2 | 0 | 6 |
| Sweden | 8^b^ | 6 | | 3 | 0 | 17 |
| Taiwan | 0 | 1 | | 0 | 0 | 1 |
| Turkey | 0 | 1 | | 4 | 0 | 5 |
| UK | 2 | 25 | | 2 | 0 | 29 |
| USA | 39^b^ | 2 | | 1 | 0 | 42 |
| Unknown | 3 | 1 | | 1 | 0 | 5 |
| Total | 74 | 70 | | 51 | 7 | 202 |

**Table S2:** **Importance of aspects considered in differential diagnosis**

^a)^ In clinicians’ questionnaire only

Percentages refer to the proportions of respondents. 7 held double specialties in neonatology and paediatric surgery. Where there were significant differences between specialties, 95% confidence intervals and a p-value are provided. Supplementary analyses showed no substantial influence from geographical variations.

Other suggested aspects to consider: Type of feed, formula/human milk (3); Previous feed history, recent change in milk feeds, speed of installation (3); Route of feeding (1); Associated illness, congenital heart disease, medication, gastroschisis and other known risk factors for NEC(4); Maternal/Prenatal history (2);Previous surgery/intervention (1); Recent blood transfusion (2); And details of clinical picture or laboratory findings(10) such as bloody stools (4), abdominal distention (3), nasogastric output/residuals (3), vomiting (1), feed Intolerance (1), abdominal pain (1), abdominal discoloration (1), acidosis (1), respiratory or circulatory instability (1), and physical exam/exam findings (2).

| Importance of aspects considered in differential diagnosis | | | | | | |
| --- | --- | --- | --- | --- | --- | --- |
|  | |  | Importance | | |  |
|  |  |  | Some | Great | |  |
|  |  |  | n [%] | n [%] | (95% CI) | p |
| "Clinical Picture", n=202 | |  | 24 [12%] | 176 [87%] |  |  |
| Radiographic findings, n=128^a^ | |  | 19 [15%] | 108 [84%] |  |  |
| Degree of prematurity, n=202 | |  | 42 [21%] | 152 [75%] |  |  |
| Age at onset, n=202 | |  | 83 [41%] | 105 [52%] |  |  |
| Laboratory findings, n=202 | |  | 105 [52%] | 73 [36%] |  |  |
|  | Neonatologists, n=77 |  | 44 [57%] | 33 [43%] | (32–54%) |  |
|  | Paediatric surgeons, n=58 |  | 23 [40%] | 32 [55%] | (42–68%) |  |
|  | Radiologists, n=74 |  | 39 [53%] | 13 [18%] | (10–28%) | <0.000 |
| Feed volumes, n=202 | |  | 113 [56%] | 38 [19%] |  |  |
| Ultrasonographic findings, n=128^a^ | |  | 59 [46%] | 37 [29%] |  |  |

**Table S3:** **Choice of modality for repeated imaging**

Percentages refer to proportions of respondents. Where there were significant differences between subgroups, 95% confidence intervals are given within brackets.

Among respondents with access to ultrasound, there was a non-significant tendency to use a different method for repeated imaging more often if NEC was not confirmed than if it was, 73 (64-80) % vs 58 (49-67) %

| Choice of modality for repeated imaging, N=202 | | | | | |  |
| --- | --- | --- | --- | --- | --- | --- |
|  |  |  | Sometimes | Always | |  |
|  |  |  | n [%] | n [%] | (95% CI) | p |
| **If NEC is not confirmed, the infant is usually examined…** | | | |  | |  |
|  | …only once | | 95 [47%] | 28 [14%] |  |  |
|  | …again, with the same method | | 123 [61%] | 55 [27%] |  |  |
|  | …again, with a different method | | 99 [49%] | 20 [10%] |  |  |
| **If NEC is confirmed, the infant is usually examined…** | | | |  | |  |
|  | …only once |  | 53 [26%] | 10 [5%] |  |  |
|  |  | Neonatologists, n=77 |  | 5 [6%] | (3−15%) |  |
|  |  | Surgeons, n=58 |  | 6 [10%] | (5−22%) |  |
|  |  | Radiologists, n=74 |  | 0 |  | 0.020 |
|  | …again, with the same method | | 75 [37%] | 107 [53%] |  |  |
|  | …again, with a different method | | 85 [42%] | 8 [4%] |  |  |
