# Supplementary material for: The role of imaging in the management of necrotising enterocolitis: a multispecialist survey and a review of the literature
Source: Eur Radiol. 2018 Mar 26;28(9):3621–31. doi: 10.1007/s00330-018-5362-x (PMC6096607; doi:10.1007/s00330-018-5362-x)
Supplement: Supplementary file 2 — (PDF 137 kb) [file 330_2018_5362_MOESM2_ESM.pdf]

The role of imaging in necrotizing enterocolitis
(for neonatologists and pediatric surgeons)

Please answer all questions according to current praxis in your department rather than to an ideal situation, except when an opinion is explicitly asked for. Answers will be handled confidentially. For administrative reasons, however, you will be asked for the name and location of your hospital. Important: use the blue button at the bottom of the page to proceed through the questionnaire and eventually submit your response so that you get a message at the end that your answers have been saved, otherwise they will not be included in the survey.

Background information

At what hospital do you work?
Please state name and location of the hospital. This question is mandatory for administrative reasons only and will not be used for identification.

[ ]

How big is the neonatal intensive care unit (NICU) of your hospital?
Please state approximate number of beds

[ ]

What is your field of expertise

- ☐ Neonatology
- ☐ Pediatric surgery

Diagnostic considerations

What differential diagnoses do you usually consider in patients with suspected NEC?

	**always**	**sometimes**	**no**
Paralytic ileus in sepsis	○	○	○
Feeding intolerance of the premature	○	○	○
Spontaneous intestinal perforation (SIP)	○	○	○
Viral enteritis	○	○	○
Gastrointestinal malformations including malrotation	○	○	○
Hirschsprung's disease	○	○	○
Ileus from meconium or other obstruction	○	○	○
Cow's milk protein allergy	○	○	○
Other. Please specify below.	○	○	○

Other:

What aspects are considered in the differential diagnosis?
Please indicate the importance of each aspect (great, some or none)

	**great**	**some**	**none**	**I don't know**
Degree of prematurity	○	○	○	○
Age of onset	○	○	○	○
Feed volumes	○	○	○	○
Radiographic findings	○	○	○	○
Ultrasonographic findings	○	○	○	○
Laboratory findings	○	○	○	○
"Clinical picture"	○	○	○	○
Other. Please specify below.	○	○	○	○

Other:

Do you use any kind of classification or staging of NEC?

	**always**	**sometimes**	**no**
Clinical appreciation of suspected, definite medical, or surgical NEC	○	○	○
Bell's criteria (or modified Bell's criteria)	○	○	○
Gordon's classification	○	○	○
Other staging or classification. Please describe below.	○	○	○

Other:

The role of imaging

How do you use imaging in the management of suspected or definite NEC?

	always	sometimes	no
For confirmation of the diagnosis			
For monitoring to stay ahead of the clinical course			
For guidance when deciding on surgery			
For guidance on when to resume feedings			
Other use. Please describe below.			

Other use:

What modality is used?
AR = Abdominal Radiography; US = Ultrasound

	always	sometimes	no
AR as first choice			
US as first choice			
AR if US is not available			
US if AR is not conclusive			
AR and US in combination			
Other routine. Please specify below			

Other routine:

How frequently are patients with confirmed or suspected NEC examined with radiological methods?

	Often	Sometimes	Rarely
More than once every 24 hours			
About once every 24 hours			
Less than once every 24 hours			

How do you decide on the frequency of radiological exams?

	always	sometimes	no
According to a fixed schedule			
According to an individual plan for each infant			
From exam to exam depending on the result and clinical course			
Other routine. Please specify below.			

Other routine:

	always	sometimes	no
Is radiation a concern			

Who decides what modality to use?

	always	sometimes	no
Clinician (neonatologist or pediatric surgeon)	○	○	○
Radiologist	○	○	○
Clinician and Radiologist by consent	○	○	○
Other routine. Please specify below.	○	○	○

Other routine:

[ ]

Repeated imaging

The word "examined" in the questions below refers only to imaging techniques, not clinical examination or chemistry.

	yes	sometimes	no	I don't know
If NEC is not confirmed, the infant is usually examined only once	○	○	○	○
If NEC is not confirmed, the infant is usually examined again with the same method	○	○	○	○
If NEC is not confirmed, the infant is usually examined again with a different method	○	○	○	○
If NEC is confirmed, the infant is usually examined only once	○	○	○	○
If NEC is confirmed, the infant is usually examined again with the same method	○	○	○	○
If NEC is confirmed, the infant is usually examined again with a different method	○	○	○	○

What is considered an indication for surgery?

AR=Abdominal radiograph; US=Ultrasound; PVG=Portal Venous Gas

	definitely	sometimes	no
Pneumoperitoneum on AR	○	○	○
Pneumoperitoneum in US	○	○	○
Portal venous gas on AR	○	○	○
Portal venous gas in US	○	○	○
“Fixed/persistent loop” on sequential AR	○	○	○
Turbid or localized peritoneal fluid in US	○	○	○
Clinical deterioration despite medical therapy	○	○	○
Other indication. Please specify below.	○	○	○

Other indication:

[ ]

Abdominal Radiography

What pictures are included in AR in NEC/suspected NEC at your department?
Vertical beam is the ordinary frontal projection with the patient in supine position. Is it done whenever NEC is suspected or are there exceptions? Horizontal beam images, used to detect free gas in the abdominal cavity, can be obtained with the infant supine, which produces a lateral projetion, or in the left decubitus position, i.e. lying on its left side, producing a frontal projection. The questions below regard both if and when horizontal beam images are used and the preferred method for obtaining them.

	in all patients	in selected patients	no	I don't know
Vertical beam	○	○	○	○
Horizontal beam (any position) first 24-48 hours	○	○	○	○
Horizontal beam (any position) after 48 hours	○	○	○	○
Horizontal beam, supine position	○	○	○	○
Horizontal beam, left decubitus position	○	○	○	○
Other routine. Please describe below.	○	○	○	○

Other routine:

Do you read the radiographs yourself

- ○ always
- ○ sometimes
- ○ no

What is evaluated in the AR in suspected NEC at your hospital?
Please indicate the importance of each finding (great, some or none).

	great	some	none	don't know
Intestinal dilatation	○	○	○	○
Pattern of gas distribution	○	○	○	○
Separation of intestinal loops	○	○	○	○
"Fixed/persistent loop" on sequential radiographs	○	○	○	○
Pneumatosis intestinalis/intramural gas	○	○	○	○
Portal venous gas	○	○	○	○
Pneumoperitoneum/free gas	○	○	○	○
Other signs. Please specify below.	○	○	○	○

Other signs:

Ultrasound

What do you think about abdominal ultrasound in NEC diagnostics?

	yes	sometimes	no	no opinion
Useful				
Readily available				
Time consuming				
Disturbs the infant				
Should be used more				

Comments:

When is ultrasound done?
AR=Abdominal Radiograph

	yes	sometimes	no
In any infant with suspected NEC			
In mild cases with inconclusive AR			
In severely ill infants with inconclusive AR/suspected but not verified perforation			
For monitoring known NEC			
Other routine. Please specify below			

Other routine:

What is evaluated with ultrasound in NEC/suspected NEC at your hospital?
If ultrasound is not used in NEC, please just indicate "no" on the first question and leave the rest blank.

	yes	no	I don't know
Ultrasound is used in NEC/suspected NEC			
Portal venous gas			
Pneumatosis intestinalis/intramural gas			
Pneumoperitoneum/free gas			
Clear/anechoic fluid			
Turbid/echoic fluid			
Focal fluid collections			
Intestinal motility			
Bowel thickening			
Bowel thinning			
Bowel perfusion (with doppler)			

	yes	no	I don't know
Mesenterical circulation (with doppler)	○	○	○
Other signs. Please specify below.	○	○	○

Other signs:

Do you and/or other colleagues at your department do ultrasound of head or abdomen yourselves?
PVG=Portal Venous Gas

	all	some	no
Head	○	○	○
Full abdominal	○	○	○
Screening for PVG	○	○	○

Comment:

Do you get support from the radiology department with reevaluation of saved cine loops or pictures?

- ☐ Yes, cineloops
- ☐ Yes, pictures
- ☐ No
- ☐ Not applicable

Would it be a good idea for neonatologists and pediatric surgeons who do not perform abdominal ultrasound today, to learn it?

- ☐ Yes, full abdominal
- ☐ Yes, screening for PVG
- ☐ No
- ☐ No opinion

Concluding comments

What would you say is the role of imaging in the management of NEC and what should it be?
Important: Even if you chose not to comment on this question, please use the blue button below to submit your response. Otherwise your answers will not be included in the survey.
When you get a message that they have been saved, you can close the browser.
