# Supplementary material for: The role of imaging in the management of necrotising enterocolitis: a multispecialist survey and a review of the literature
Source: Eur Radiol. 2018 Mar 26;28(9):3621–31. doi: 10.1007/s00330-018-5362-x (PMC6096607; doi:10.1007/s00330-018-5362-x)
Supplement: Supplementary file 3 — (PDF 166 kb) [file 330_2018_5362_MOESM3_ESM.pdf]

The role of imaging in necrotizing enterocolitis – radiologists' version

Please answer all questions according to current praxis in your department rather than to an ideal situation, except when an opinion is explicitly asked for. Answers will be handled confidentially. For administrative reasons, however, you will be asked for the name and location of your hospital. Important: use the blue button at the bottom of the page to proceed through the questionnaire and eventually submit your response so that you get a message at the end that your answers have been saved, otherwise they will not be included in the survey.

Background information

At what hospital do you work?
Please state name and location of the hospital. The question is mandatory for administrative reasons only and will not be used for identification.

How big is the neonatal intensive care unit (NICU) of your hospital?
Please state approximate number of beds if you know it.

What is your field of expertise

- [ ] General radiology
- [ ] Pediatric radiology

Diagnostic considerations

What differential diagnoses come to mind when you encounter a request for imaging with respect to NEC?
For considerations entirely left to the clinician, please choose the response option "no".

	always	sometimes	no
Paralytic ileus in sepsis	○	○	○
Spontaneous intestinal perforation (SIP)	○	○	○
Gastrointestinal malformations including malrotation	○	○	○
Hirschsprung’s disease	○	○	○
Ileus from meconium or other obstruction	○	○	○
Gastroenteritis	○	○	○
Other. Please specify below.	○	○	○

Other/comments:

Which clinical information do you consider relevant in order to evaluate the request and/or the study?
Please indicate the importance of each aspect (great, some or none)

	great	some	none	no opinion
Degree of prematurity	○	○	○	○
Age of onset	○	○	○	○
Feed volumes	○	○	○	○
Laboratory findings	○	○	○	○
“Clinical picture”	○	○	○	○
Other. Please specify below.	○	○	○	○

Other aspect/comment:

Is any classification or staging of NEC used in the communication between radiologists and clinicians at your hospital?

	always	sometimes	no
Clinical appreciation of suspected, definite medical, or surgical NEC	○	○	○
Bell’s criteria (or modified Bell's criteria)	○	○	○
Gordon’s classification	○	○	○
Other staging or classification. Please describe below.	○	○	○

Other:

The role of imaging

How is imaging used in the management of suspected or definite NEC?

	always	sometimes	no	I don't know
For confirmation of the diagnosis	○	○	○	○
For monitoring to stay ahead of the clinical course	○	○	○	○
For guidance when deciding on surgery	○	○	○	○
For guidance on when to resume feedings	○	○	○	○
Other use. Please describe below.	○	○	○	○

Other use:

What modality is used
AR = Abdominal Radiography; US = Ultrasound

	always	sometimes	no
AR as first choice	○	○	○
US as first choice	○	○	○
AR if US is not available	○	○	○
US if AR is not conclusive	○	○	○
AR and US in combination	○	○	○
Individualized approach	○	○	○
Other routine. Please specify below.	○	○	○

Other routine:

Who decides what modality to use?

	always	sometimes	no
Clinician (neonatologist or pediatric surgeon)	○	○	○
Radiologist	○	○	○
Clinician and Radiologist by consent	○	○	○
Other routine. Please specify below.	○	○	○

Other routine:

How frequently are patients with confirmed or suspected NEC examined with radiological methods?

	often	sometimes	rarely	I don't know
More than once every 24 hours	○	○	○	○
About once every 24 hours	○	○	○	○
Less than once every 24 hours	○	○	○	○

How is the frequency of radiological exams decided?

	always	sometimes	no
According to a fixed schedule. Please specify below.	○	○	○
According to an individual plan for each infant	○	○	○
From exam to exam depending on the result	○	○	○
Other routine. Please specify below.	○	○	○

Description of fixed schedule or other routine:

	always	sometimes	no
Is radiation a concern	○	○	○

Repeated imaging

The word "examined" in the questions below refers only to imaging techniques, not clinical examination or chemistry.

	yes	sometimes	no	I don't know
If NEC is not confirmed, the infant is usually examined only once	○	○	○	○
If NEC is not confirmed, the infant is usually examined again with the same method	○	○	○	○
If NEC is not confirmed, the infant is usually examined again with a different method	○	○	○	○
If NEC is confirmed, the infant is usually examined only once	○	○	○	○
If NEC is confirmed, the infant is usually examined again with the same method	○	○	○	○
If NEC is confirmed, the infant is usually examined again with a different method	○	○	○	○

Which of the following findings are serious enough to influence the decicion for surgery?
AR=Abdominal Radiograph; US=Ultrasound

	definitely	sometimes	no	I don't know
Pneumoperitoneum on AR	○	○	○	○
Pneumoperitoneum in US	○	○	○	○
Portal venous gas on AR	○	○	○	○
Portal venous gas in US	○	○	○	○
“Fixed/persistent loop” on sequential AR	○	○	○	○
Turbid or localized peritoneal fluid in US	○	○	○	○
Other. Please specify below.	○	○	○	○

Other:

Abdominal Radiography

What pictures are included in the abdominal radiography in NEC/suspected NEC at your department?

Vertical beam is the ordinary frontal projection with the patient in supine position. Is it done whenever NEC is suspected or are there exceptions? Horizontal beam images, used to detect free gas in the abdominal cavity, can be obtained with the infant supine, which produces a lateral projetion, or in the left decubitus position, i.e. lying on its left side, producing a frontal projection. The questions below regard both if and when horizontal beam images are used and the preferred method for obtaining them.

	in all patients	in selected patients	no	I don't know
Vertical beam	○	○	○	○
Horizontal beam (any position) first 24-48 hours	○	○	○	○
Horizontal beam (any position) after 48 hours	○	○	○	○
Horizontal beam, supine position	○	○	○	○
Horizontal beam, left decubitus position	○	○	○	○
Other routine. Please describe below	○	○	○	○

Other routine:

[ ]

Do the neonatologists read the radiographs themselves?

○ always

○ sometimes

○ no

○ I don't know

What is evaluated in the AR in suspected NEC at your department?
Please indicate the importance of each finding (great, some or none).

	great	some	none
Intestinal dilatation	○	○	○
Pattern of gas distribution	○	○	○
Separation of intestinal loops	○	○	○
"Fixed/persisting loop" on sequential radiographs	○	○	○
Pneumatosis intestinalis/intramural gas	○	○	○
Portal venous gas	○	○	○
Pneumoperitoneum/free gas	○	○	○
Other signs. Please specify below.	○	○	○

Other signs:

[ ]

Ultrasound

What do you think about abdominal ultrasound in NEC diagnostics?

	yes	sometimes	no	no opinion
Useful	○	○	○	○
Readily available	○	○	○	○
Time consuming	○	○	○	○
Disturbs the infant	○	○	○	○
Should be used more	○	○	○	○

Comments:

When is ultrasound done?
AR=Abdominal Radiograph

	yes	sometimes	no
In any infant with suspected NEC	○	○	○
In mild cases with inconclusive AR	○	○	○
In severely ill infants with inconclusive AR/suspected but not verified perforation	○	○	○
For monitoring known NEC	○	○	○
Other routine. Please specify below	○	○	○

Other routine:

What is evaluated with ultrasound in NEC/suspected NEC at your hospital?
If ultrasound is not used in NEC, indicate "no" on the first question and leave the rest blank.

	yes	no	I don't know
Ultrasound is used in NEC/suspected NEC	○	○	○
Portal venous gas	○	○	○
Pneumatosis intestinalis/intramural gas	○	○	○
Pneumoperitoneum/free gas	○	○	○
Clear/anechoic fluid	○	○	○
Turbid/echoic fluid	○	○	○
Focal fluid collections	○	○	○
Intestinal motility	○	○	○
Bowel thickening	○	○	○
Bowel thinning	○	○	○
Bowel perfusion (with doppler)	○	○	○
Mesenterical circulation (with doppler)	○	○	○

	yes	no	I don't know
Other signs. Please specify below.	○	○	○

Other signs:

Do neonatologists or pediatric surgeons at your hospital do ultrasound of head or abdomen themselves?

	all	some	no
Head	○	○	○
Full abdominal	○	○	○
Screening for PVG	○	○	○

Comment:

Are saved pictures or cine loops made by neonatologists reevaluated by a radiologist??

- ☐ Yes, cineloops
- ☐ Yes, pictures
- ☐ No
- ☐ Not applicable

Would it be a good idea for neonatologists and pediatric surgeons who do not perform abdominal ultrasound today, to learn it?

- ☐ Yes, full abdominal
- ☐ Yes, screening for PVG
- ☐ No
- ☐ No opinion

Concluding comments

What would you say is the role of imaging in the management of NEC and what should it be? Important: Even if you chose not to comment on this question, please use the blue button below to submit your response. Otherwise your answers will not be included in the survey. When you get a message that they have been saved, you can close the browser.
